# Temporary Esophageal Exclusion With Absorbable Ligatures: A Salvage Strategy for Pediatric Button Battery-Related Perforation

**DOI:** 10.7759/cureus.113261

**Published:** 2026-07-23

**Authors:** Despina Panayiotou, Elisavet Kanna, Zoi Lamprinou, Ioanna Argyri, Ioannis Skondras

**Affiliations:** 1 Otolaryngology Department, Panagiotis & Aglaia Kyriakou Children's Hospital, Athens, GRC; 2 Second Pediatric Surgery Department, Panagiotis & Aglaia Kyriakou Children's Hospital, Athens, GRC; 3 Pediatric Gastroenterology Department, Panagiotis & Aglaia Kyriakou Children's Hospital, Athens, GRC

**Keywords:** absorbable ligatures, button battery ingestion, esophageal diversion, esophageal perforation, pediatric surgery, temporary esophageal exclusion

## Abstract

Button battery ingestion constitutes a life-threatening pediatric emergency, particularly when impacted in the esophagus, causing rapid tissue necrosis. Esophageal perforation is among its most severe sequelae, yet optimal management remains unclear. Organ-preserving strategies are especially important in children but are rarely reported. We report a case of a six-year-old boy who developed distal esophageal perforation following button battery impaction. Initial endoscopic removal and conservative management were insufficient, necessitating subsequent thoracotomy, loop cervical esophagostomy, and Stamm gastrostomy. Persistent bilious drainage from the esophagostomy and chest drains prompted a salvage technique using proximal and distal absorbable ligatures to exclude the injured esophagus. This achieved rapid sepsis control while preserving the native esophagus. Spontaneous recanalization occurred approximately two months after ligature placement, following their gradual dissolution, restoring continuity without formal reanastomosis. This case suggests that temporary esophageal exclusion with absorbable ligatures is a feasible, simple, and organ-preserving salvage option, expanding the therapeutic armamentarium for pediatric surgeons managing complex esophageal perforations after button battery ingestion.

## Introduction

Button battery ingestion represents one of the most high-risk pediatric foreign body emergencies, accounting for up to one-quarter of cases and increasing in frequency due to the widespread use of household electronics [1-3]. When lodged in the esophagus, button batteries can cause rapid corrosive injury, with full-thickness necrosis developing within hours due to electrolysis [4]. The absence of a serosal layer renders the esophagus particularly susceptible to life-threatening complications, with reported pediatric mortality rates up to 30% [5]. Major sequelae include esophageal perforation, tracheoesophageal fistula, and aortoesophageal fistula [2]. Therefore, urgent endoscopic removal, ideally within two hours of ingestion, is essential to reduce the risk of catastrophic injury [1-3,6].

Management of esophageal perforation remains challenging, with no universally accepted gold standard [7]. Therapeutic options range from conservative to surgical, depending on the extent of injury and clinical stability. In children, conservative management is often attempted initially; however, surgical intervention becomes necessary if deterioration, uncontrolled sepsis, or persistent leakage occurs [5,8,9]. Surgical options include primary repair, diversion or exclusion procedures when primary repair is not feasible, resection for extensive tissue destruction, and, in selected stable patients, endoscopic approaches [6,10,11].

We report the case of a six-year-old boy with distal esophageal perforation following button battery impaction. Initial conservative treatment and subsequent diversion with loop esophagostomy were unsuccessful, as persistent bilious drainage from both the esophagostomy and chest drains indicated ongoing contamination. A salvage procedure employing temporary esophageal exclusion with proximal and distal absorbable ligatures achieved complete isolation of the injured segment and rapid control of sepsis. Spontaneous recanalization occurred as the ligatures dissolved, restoring luminal continuity without the need for formal reanastomosis. This case demonstrates a rare pediatric application of a previously described temporary esophageal exclusion technique and underscores its potential as a relatively simple, organ-preserving salvage strategy in complex pediatric esophageal perforations.

## Case presentation

**Figure 1:**
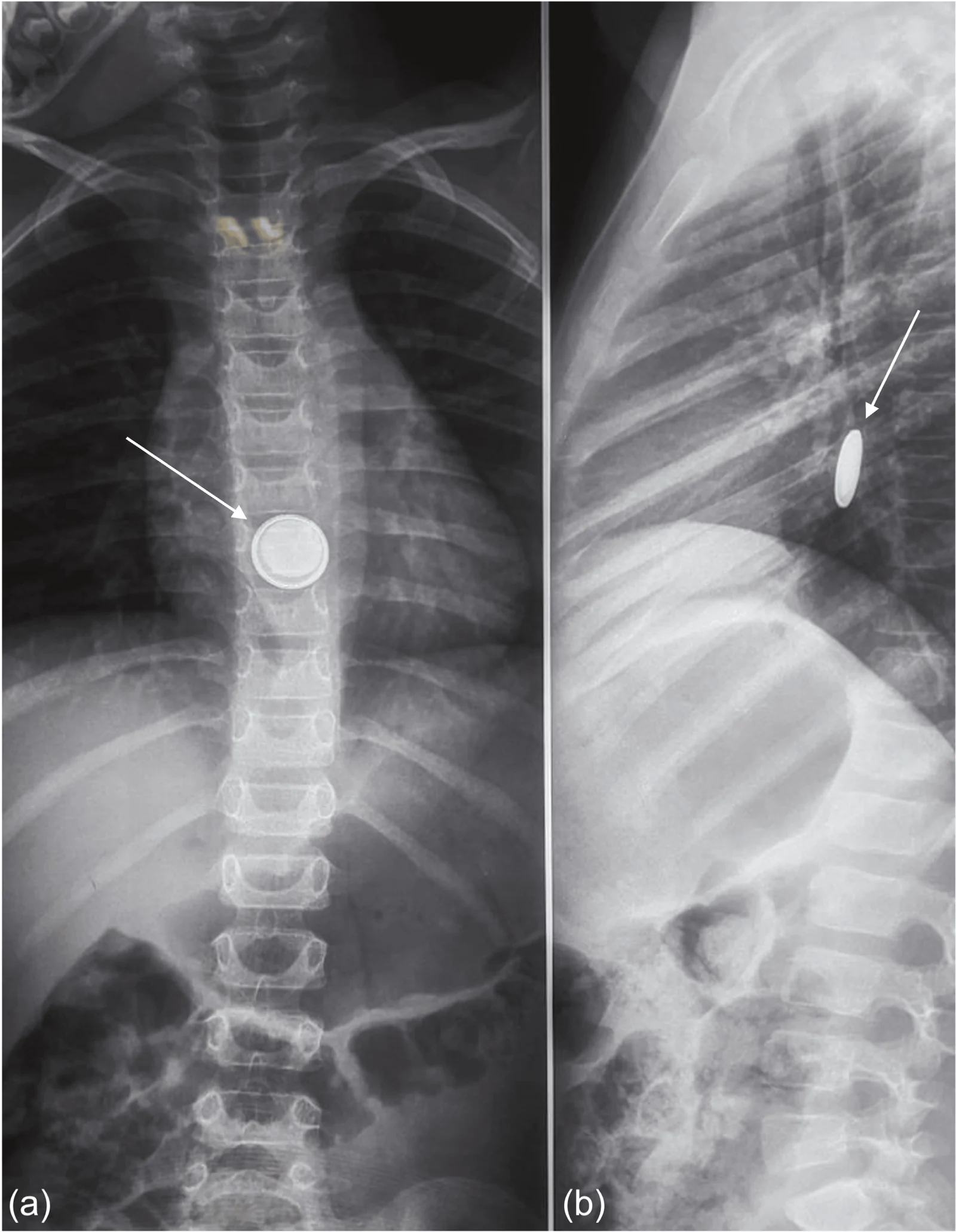
Plain radiographs demonstrating a button battery impacted in the distal esophagus. (a) Anteroposterior view showing the button battery lodged in the distal esophagus. (b) Lateral view confirming the esophageal location of the foreign body. White arrows indicate the impacted button battery.

**Figure 2:**
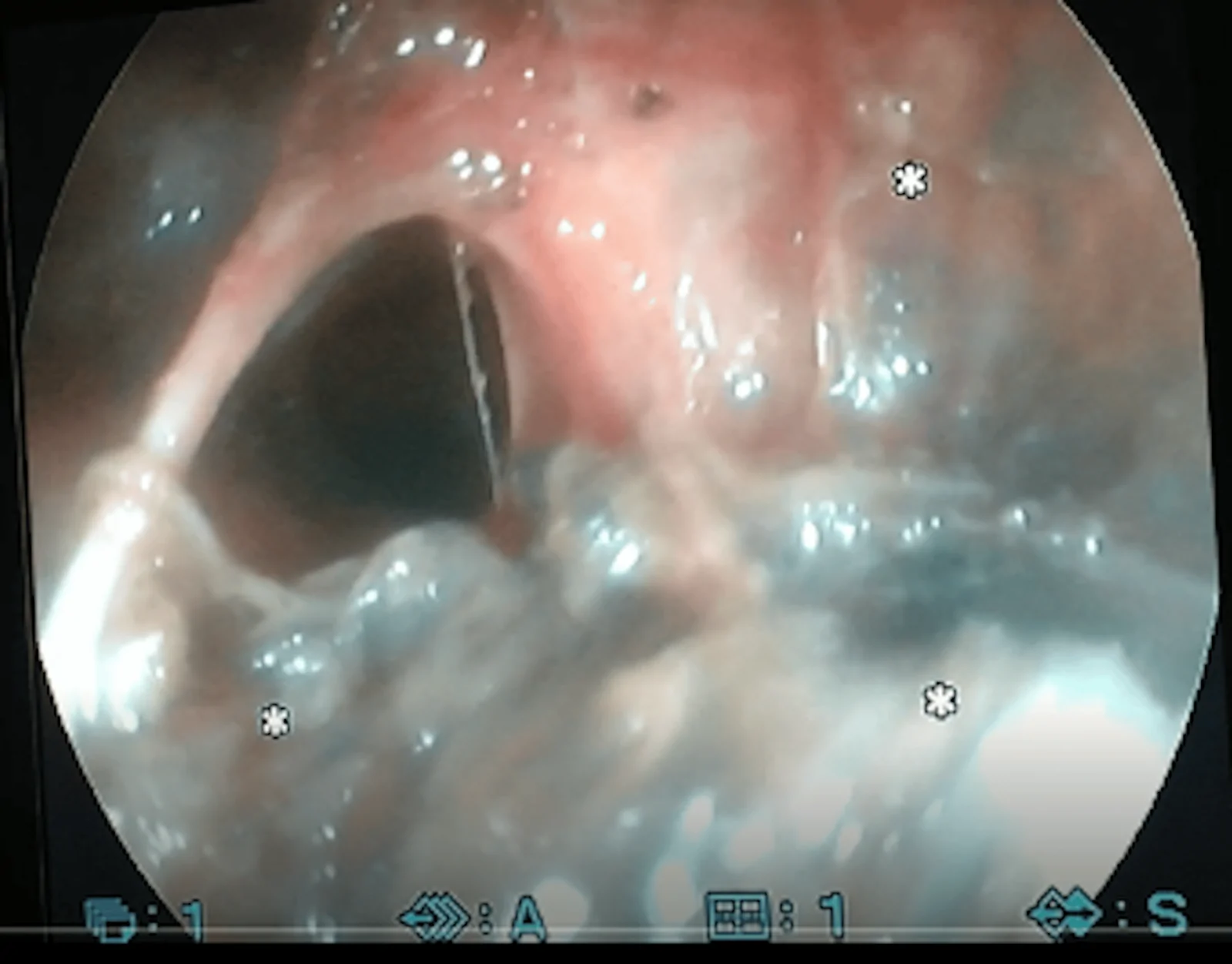
Endoscopy view demonstrating extensive necrosis of the esophageal mucosa with two submucosal tunnels, findings consistent with a full-thickness esophageal rupture.

**Figure 3:**
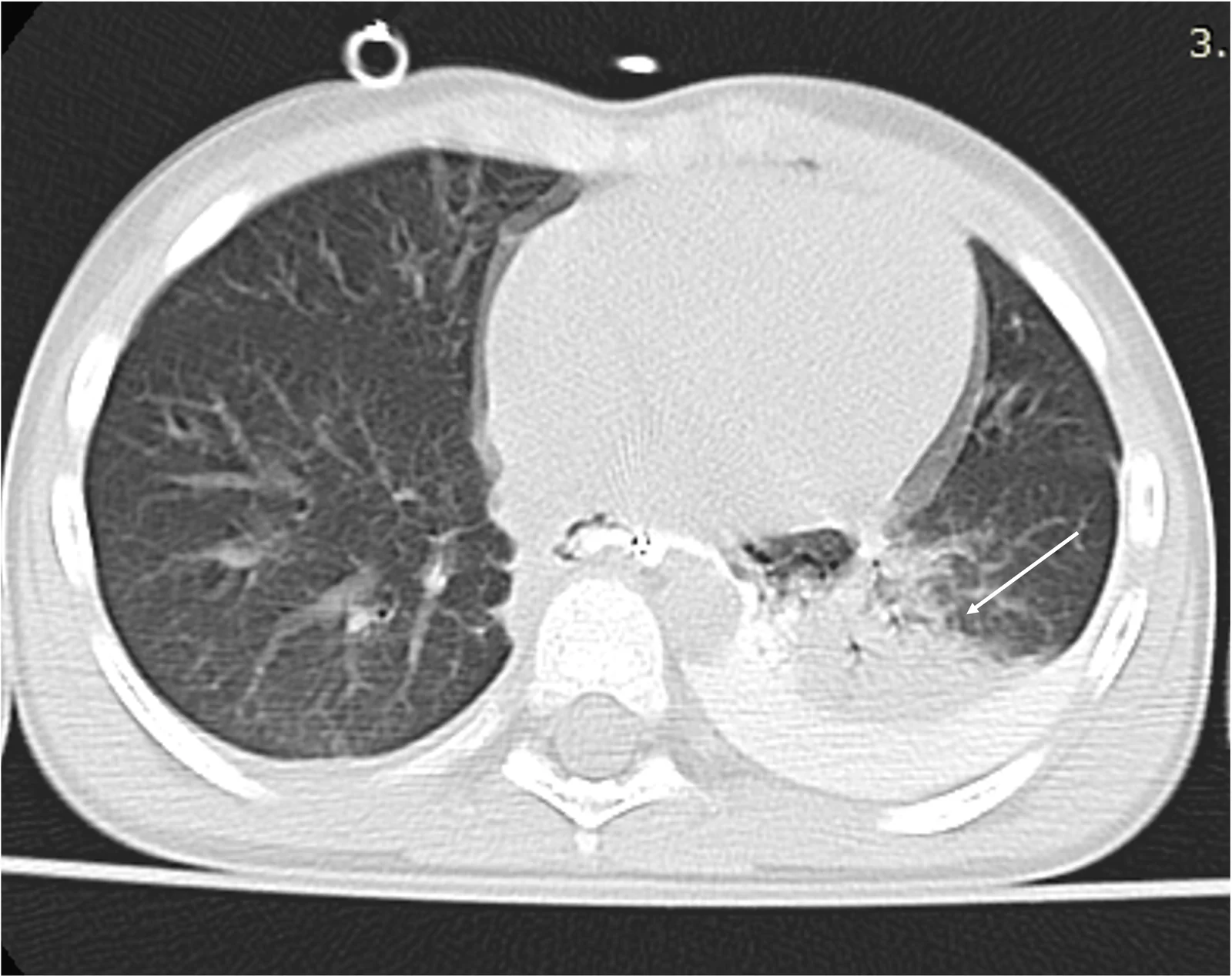
Axial thoracic CT scan showing a left pleural effusion and mediastinal inflammatory changes. The white arrow indicates the area of mediastinal inflammation adjacent to the distal esophageal rupture. CT: computed tomography

A previously healthy six-year-old boy, weighing 24 kg, presented to our emergency department with retrosternal burning pain following button battery ingestion. Plain radiographs demonstrated impaction of the battery in the distal esophagus (Figures 1a, 1b). Urgent endoscopy, performed within four hours, revealed extensive mucosal necrosis and two submucosal tunnels (Figure 2). The battery was advanced into the stomach; however, initial retrieval was unsuccessful. Contrast esophagogram confirmed distal esophageal perforation, while computed tomography revealed mediastinal leakage and pleural effusion (Figure 3). A chest tube was inserted, immediately draining 170 mL of serous fluid, and a repeat endoscopy successfully removed the battery. The patient was managed in the pediatric intensive care unit, with intravenous antibiotics, proton pump inhibitors, and corticosteroids.

**Figure 4:**
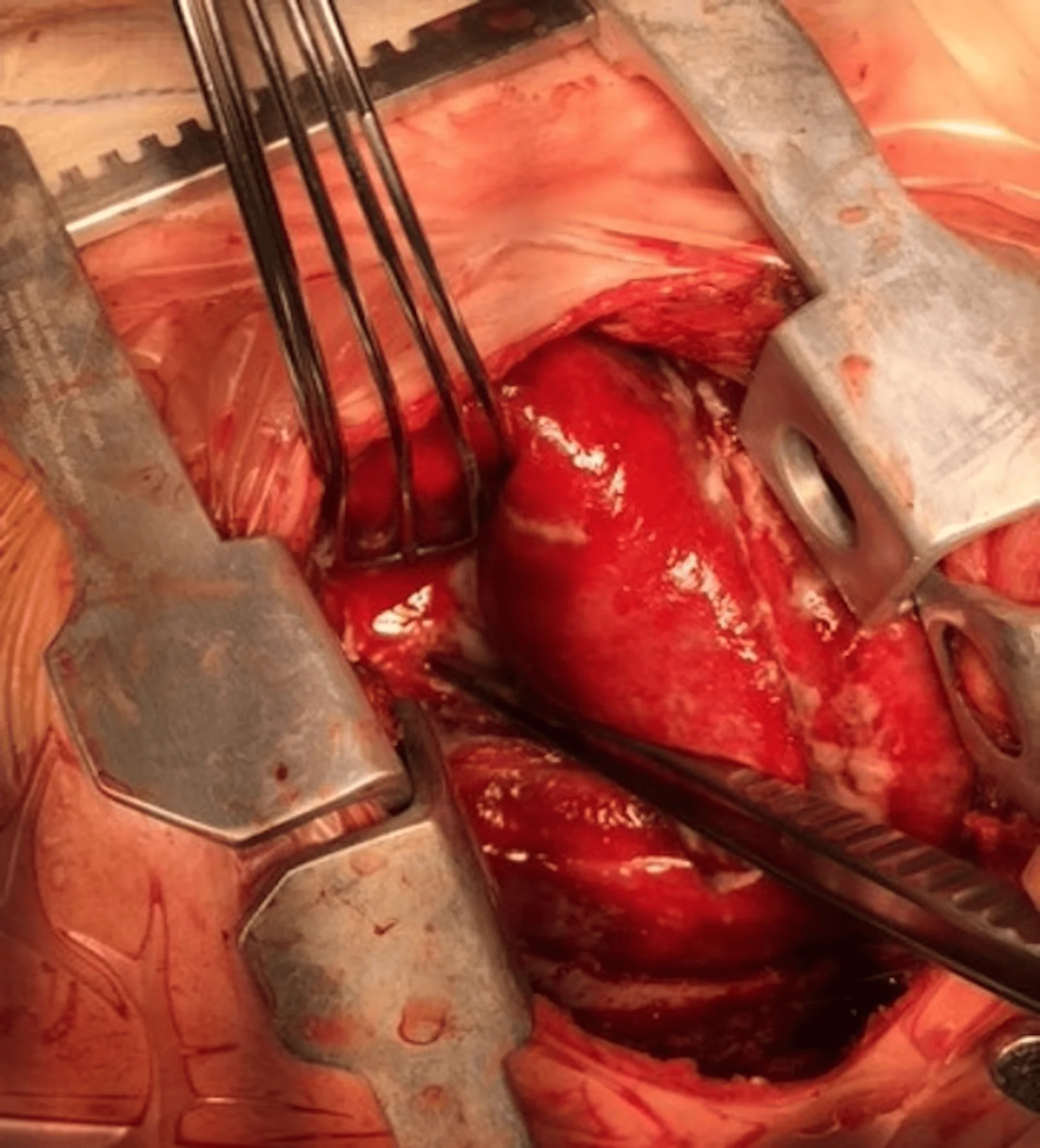
Intraoperative photograph during thoracotomy, with DeBakey forceps pointing to the site of esophageal perforation.

**Figure 5:**
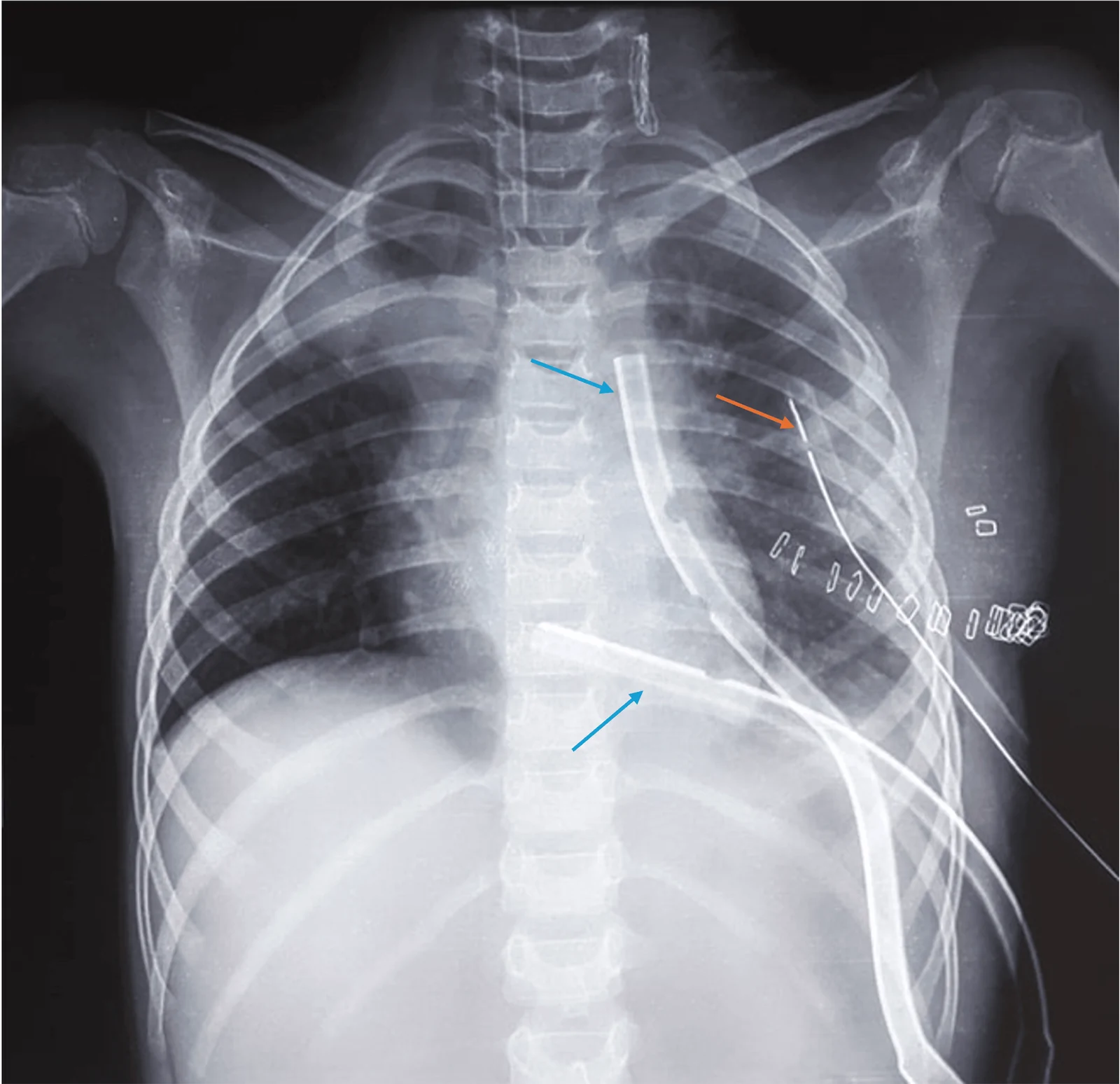
Postoperative chest radiograph showing one left thoracic drain (orange arrow) and two mediastinal drains (blue arrows).

He remained intubated for 48 hours, and within 24 hours of extubation, he developed severe respiratory deterioration associated with bilateral pleural effusions. Thoracic ultrasonography showed air bubbles within the pleural fluid, raising suspicion of ongoing esophageal leakage. Reintubation was required, after which the chest tube drained thick purulent material. Although the patient initially stabilized, persistent respiratory compromise, bilateral pleural collections, and purulent drainage prompted surgical intervention. Thoracotomy was performed, with loop cervical esophagostomy and Stamm gastrostomy, alongside mediastinal drains (Figures 4, 5).

**Figure 6:**
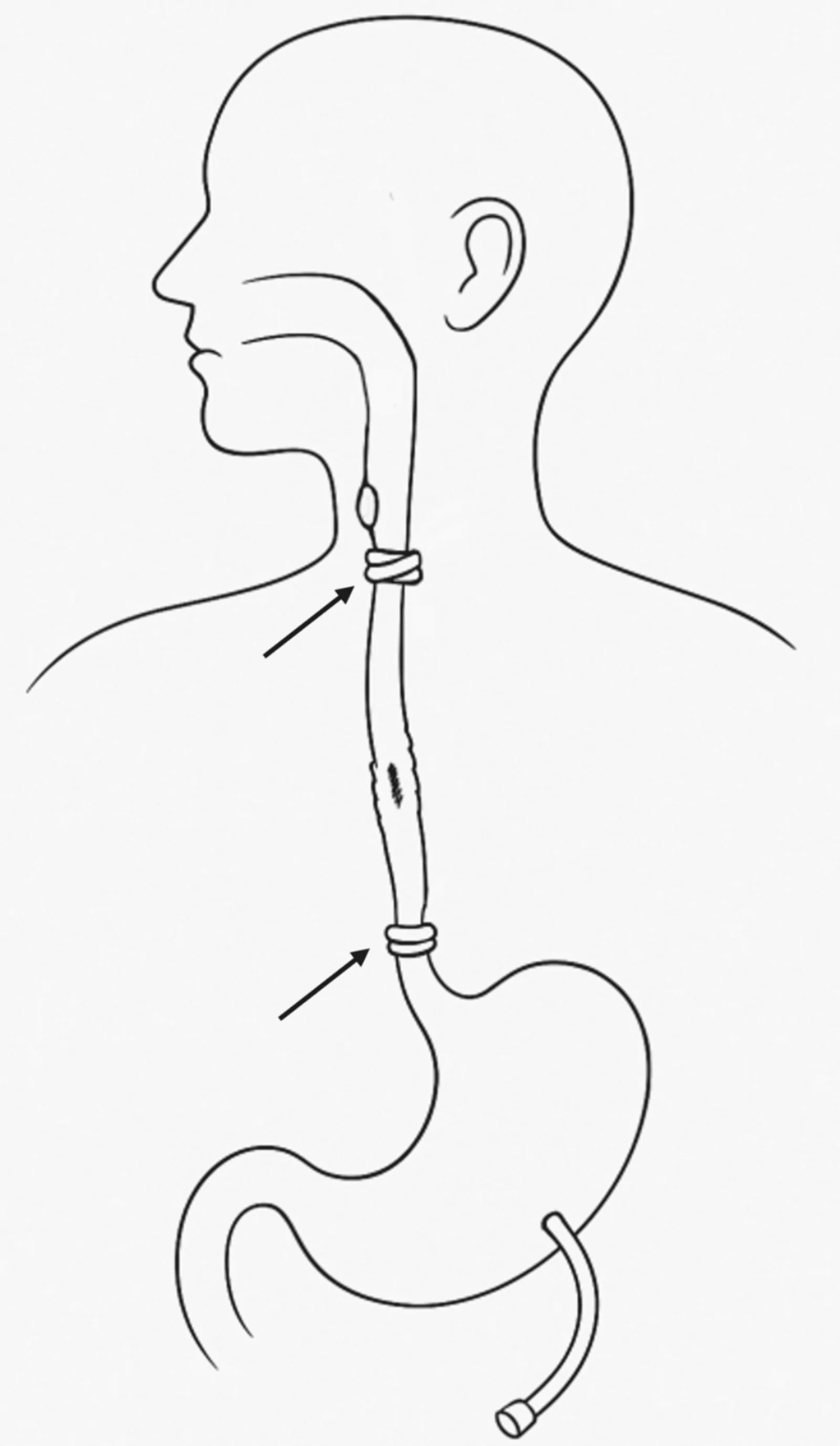
Schematic of temporary esophageal exclusion using absorbable ligatures. Proximal and distal absorbable ligatures (black arrows) were placed just below the cervical esophagostomy and at the esophagogastric junction, respectively. This created complete isolation of the injured thoracic esophageal segment, while the gastrostomy tube provided gastric decompression and enteral access until spontaneous recanalization occurred. Image credit: The figure was created by the author Despina Panayiotou using Notes on iPad (Apple Inc., Cupertino, CA)

Despite cervical esophageal diversion, bilious output persisted from both the esophagostomy and chest drains, indicating ongoing contamination of the injured esophageal segment. As a salvage procedure, No. 2 absorbable multifilament braided ligatures were placed at the esophagogastric junction and just below the cervical esophagostomy. This maneuver created complete proximal and distal isolation of the perforated esophagus (Figure 6).

This resulted in a rapid reduction of drainage output, resolution of sepsis, and progressive clinical improvement. After several weeks of parenteral nutrition and gradual recovery, the drains were removed, and the patient was discharged.

A follow-up contrast study two months later demonstrated brisk passage of contrast into the stomach, confirming spontaneous recanalization of the previously excluded esophagus and restoring luminal continuity without formal reanastomosis. A focal narrowing at the T9 level was also identified, for which serial postoperative balloon dilatations were performed through the distal limb of the loop esophagostomy.

**Figure 7:**
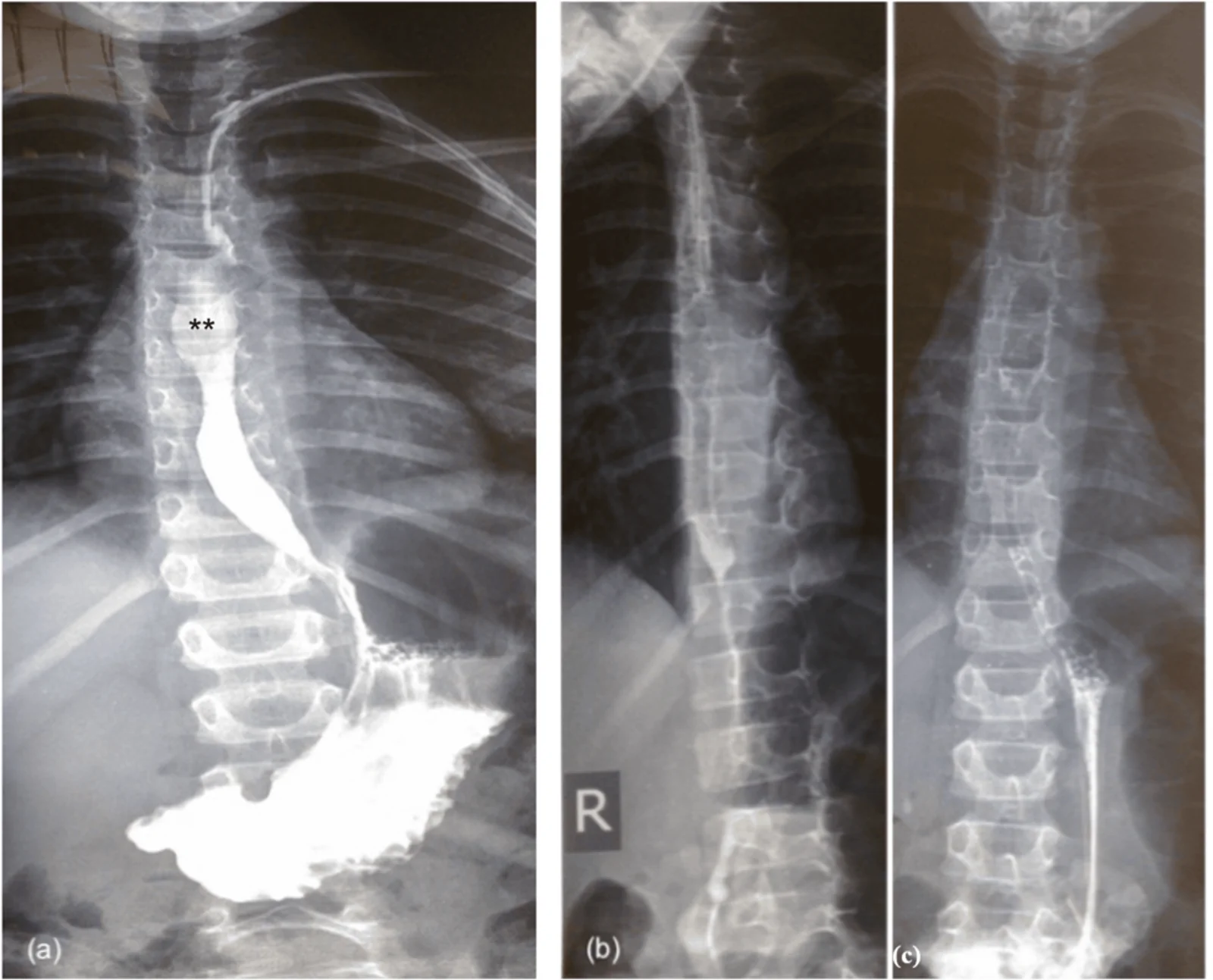
Postoperative esophagograms following temporary esophageal exclusion. (a) Contrast study performed before esophagostomy closure demonstrating passage of contrast into the stomach. The double asterisk indicates mild mid-esophageal dilatation distal to the tracheal bifurcation. (b,c) Sequential images obtained during the contrast study after esophagostomy closure demonstrating uninterrupted passage of contrast into the stomach without evidence of leak or significant stricture.

The patient was later readmitted for elective esophagostomy closure. Repeat contrast study showed no stricture or leak, with only mild mid-esophageal dilatation below the tracheal bifurcation (Figure 7a). Following esophagostomy closure, a contrast study demonstrated uninterrupted passage of contrast into the stomach, without evidence of a leak or significant stricture (Figure 7b). He subsequently resumed oral intake and was discharged in good condition. At 12 months, he remained clinically well, with no additional complications documented. No further hospital presentations related to the esophageal injury occurred over the following eight years.

**Figure 8:**
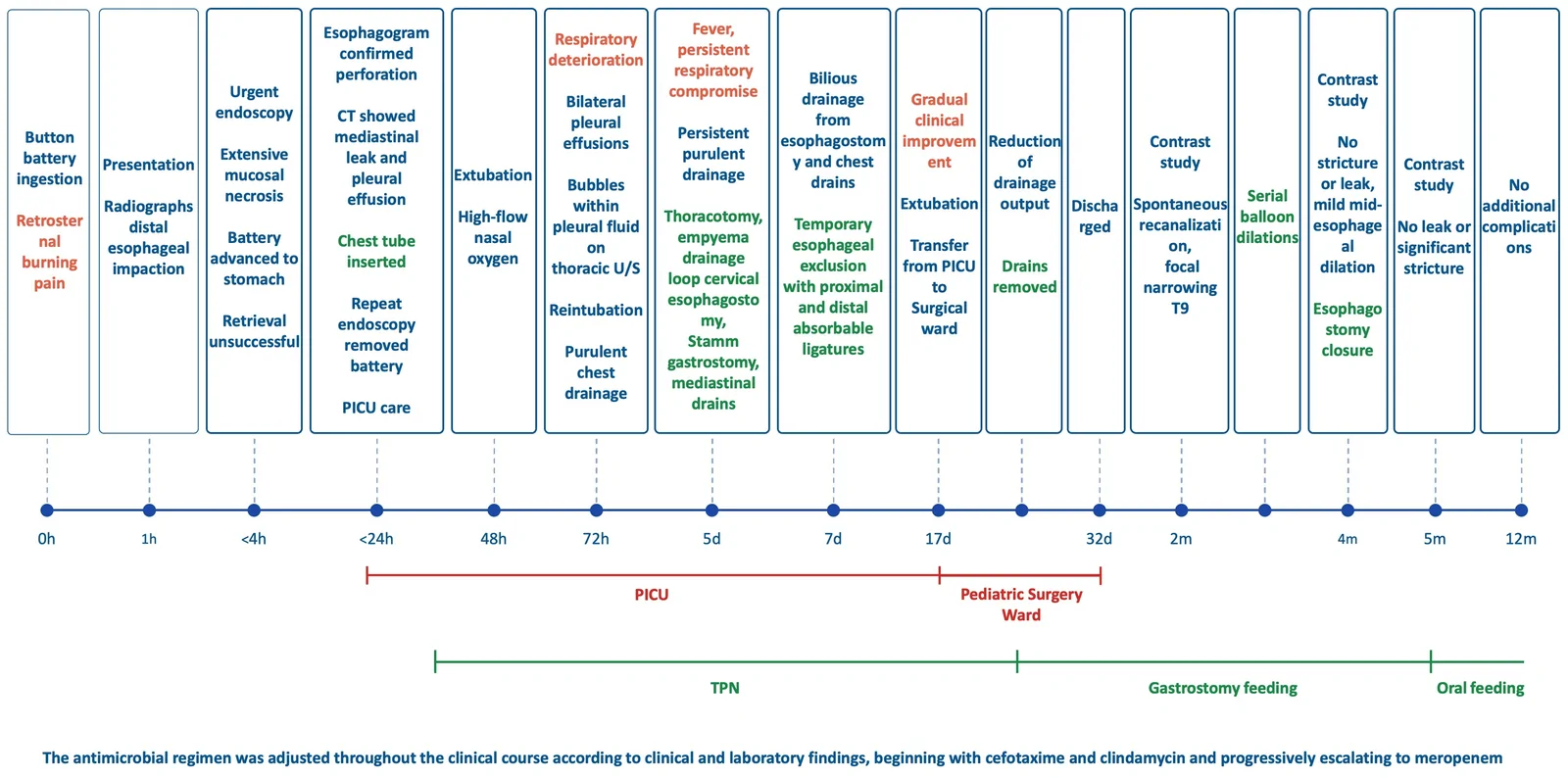
Timeline of the patient’s clinical course. Major clinical and radiological findings, interventions, and recovery milestones are summarized in chronological order. PICU: pediatric intensive care unit; TPN: total parenteral nutrition; CT: computed tomography; U/S: ultrasonography Image credit: The figure was created by the authors using Microsoft PowerPoint (Microsoft Corporation, Redmond, WA)

The timeline of the patient’s clinical course is summarized in Figure 8.

## Discussion

Button battery ingestion poses a significant risk to the pediatric population, with cases increasing worldwide owing to the widespread use of button batteries in household electronic devices [1-3]. Recent evidence suggests that this rising incidence has been accompanied by a sevenfold increase in the relative risk of severe morbidity over the past two decades [12]. When lodged in the esophagus, button batteries can cause rapid tissue necrosis, often beginning within one to two hours, potentially progressing to severe complications [4,13]. Clinical outcomes are strongly influenced by the timing of intervention [1,3,4,13]. Once esophageal button battery impaction is identified, prompt endoscopic removal is imperative. Current World Society of Emergency Surgery (WSES) and European Society for Pediatric Gastroenterology, Hepatology and Nutrition guidelines recommend emergent endoscopy within two hours and no later than six hours to minimize the risk of severe injury [6,12]. During removal, careful inspection of the esophageal mucosa is essential for assessing the extent of injury and identifying early complications [1].

Esophageal perforation is among the most serious complications of button battery ingestion. Although relatively uncommon in children [9], it represents a life-threatening emergency and most frequently involves the intrathoracic esophagus [10,11]. In this setting, management strategies vary widely, ranging from conservative treatment to extensive surgical intervention, and no universally accepted gold standard has been established. Treatment should therefore be individualized, guided by the patient’s clinical status, the extent of tissue injury, and the presence of associated complications [5,7,10,14].

Although operative closure is typically favored in adults, pediatric esophageal perforation is more commonly managed nonoperatively [5,8]. Conservative management typically includes cessation of oral intake, gastric decompression, antibiotic and acid-suppression therapy, adequate drainage, and total parenteral nutrition when required [3,8,9]. This approach may be appropriate for clinically stable patients with contained perforations and limited contamination, provided close monitoring is ensured [6]. Martinez et al. [8] demonstrated successful nonoperative management even in extensive pediatric perforations, while both their findings and those of Kimball et al. [3] emphasized that surgical intervention should be reserved for patients in whom conservative measures fail or when the defect persists or worsens. Nevertheless, nonoperative treatment is not universally successful, and clinical deterioration may require delayed surgery. In the present case, persistent mediastinal leakage, progressive pleural contamination, and subsequent clinical sepsis despite initial conservative management necessitated surgical intervention.

Surgery remains central to the management of esophageal perforation [11]. In pediatric patients, reported operative approaches include primary repair, diversion or exclusion procedures, esophageal resection, and endoscopic techniques [5,11].

Primary repair consists of debridement, layered closure, and reinforcement with vascularized flaps when feasible. In adults, it has been associated with the lowest mortality, even in delayed presentations [5,10]. Pediatric outcomes are also favorable in selected cases. In a series of 24 pediatric patients with esophageal perforation, most were successfully managed with primary closure [9]. Similarly, Peters et al. [7] reported successful foreign body removal and primary repair of the defect during the same procedure after failed rigid esophagoscopy.

According to the WSES guidelines, esophageal diversion or exclusion may be considered when direct repair is not feasible, in a manner analogous to a colostomy for distal colonic injury, to prevent ongoing contamination [6]. Diversion is particularly indicated in hemodynamically unstable patients, in cases of friable esophageal tissue, or when extensive mediastinitis precludes safe closure [5,11]. In these circumstances, complete diversion serves as a salvage strategy to control intrathoracic contamination and sepsis, and is commonly achieved through a cervical esophagostomy combined with a gastrostomy or a jejunostomy [6,15].

Complete diversion can be achieved with end-esophagostomy; however, subsequent restoration of esophageal continuity is often challenging and may require gastric or colonic interposition. Conversely, loop esophagostomies preserve the native esophagus and are more readily reversible, yet incomplete diversion may allow persistent mediastinal contamination by oral secretions [15].

To overcome this limitation, temporary esophageal exclusion using absorbable ligatures has been described to achieve complete diversion while preserving the native esophagus. In a series of five patients, Koniaris et al. [15] demonstrated that placement of a distal absorbable ligature beyond a loop esophagostomy was associated with no leaks or strictures during four years of follow-up. Double esophageal exclusion was first described in 1956 and later refined to reduce the need for reconstructive surgery. The technique involves placement of absorbable ligatures distal to the cervical esophagostomy, to divert oral secretions, and at the esophagogastric junction, to prevent reflux, thereby allowing effective control of leakage and sepsis. As the ligatures dissolve spontaneously after approximately four weeks, esophageal continuity may be restored without reoperation [9,15,16]. Chang et al. [16] described seven adult patients treated with a one-stage approach that combined primary closure, cervical T-tube esophagostomy, and total esophageal exclusion with absorbable ligatures, all of whom achieved luminal recanalization without reoperation.

In our patient, we applied the same principle of temporary total esophageal exclusion using absorbable ligatures. Unlike the one-stage procedure described by Chang et al., esophageal exclusion was performed as a staged salvage intervention after persistent bilious output from both the esophagostomy and chest drains. This strategy achieved effective control of sepsis while preserving the native esophagus, underscoring the adaptability of this rarely used adult technique to complex pediatric injuries.

A similar principle is applied in duodenal injuries, where temporary diversion protects a fragile repair. Alsaadi et al. [17] reported primary duodenal closure with pyloric exclusion using absorbable ligatures, thereby diverting gastric contents and allowing healing without resection. Spontaneous pyloric reopening occurred as the ligatures dissolved, restoring gastrointestinal continuity without reoperation. This approach parallels temporary esophageal exclusion, as both employ controlled, reversible diversion to limit contamination, preserve native tissue, and permit recovery before spontaneous restoration of luminal continuity.

Other surgical options include esophageal resection, which is generally reserved as a last-resort strategy, typically in the setting of delayed presentation, extensive tissue necrosis, advanced mediastinitis, or hemodynamic instability [6,11,14]. In pediatric patients, resection is rarely required, as most perforations can be managed with esophagus-preserving strategies [9].

Finally, endoscopic therapy has gained an increasingly important role in the management of esophageal perforation. In line with contemporary series evaluating multimodal treatment strategies [14], endoscopy is recommended as the first-line modality for diagnosis and initial management; however, its therapeutic role is limited to early diagnosed, hemodynamically stable, nonseptic patients, particularly those with small iatrogenic perforations or high surgical risk. In cases of extensive mediastinal contamination, surgical intervention should not be delayed. Endoscopic treatment options include clips, stents, suturing, and endoscopic vacuum-assisted closure [10,11].

Close follow-up is essential even after clinical recovery. Contrast study or endoscopy within the first four weeks is strongly recommended to rule out esophageal stenosis, the most common complication following button battery ingestion [1].

## Conclusions

This case underscores the importance of a stepwise, individualized approach to pediatric esophageal perforation following button battery ingestion, with escalation from conservative management to surgical intervention when initial measures fail. Temporary esophageal exclusion using proximal and distal absorbable ligatures allowed complete isolation of the injured segment and proved to be an effective salvage technique. In our patient, this approach achieved control of ongoing mediastinal contamination and sepsis after failure of conservative and diversion measures, while preserving the native esophagus. Spontaneous restoration of esophageal continuity occurred as the ligatures dissolved, without the need for formal reconstructive surgery. In the absence of a universally accepted treatment protocol, the outcome of this case suggests that temporary esophageal exclusion may offer a relatively simple, reversible, and organ-preserving salvage option, expanding the pediatric surgical armamentarium for managing severe esophageal perforation. However, the evidence remains limited to case reports and small case series, precluding definitive conclusions. Further clinical evidence is needed to better define the indications, safety, effectiveness, and long-term outcomes of this technique.
